# Synergistic protective and regenerative effects of hyaluronic acid and polynucleotides against UVA-induced oxidative stress in dermal fibroblasts

**DOI:** 10.1038/s41598-026-37730-5

**Published:** 2026-01-30

**Authors:** Trang Thanh Thien Tran, Soon Chul Heo, Jun Hee Lee, Hae-Won Kim

**Affiliations:** 1 Institute of Tissue Regeneration Engineering (ITREN), Dankook University, Cheonan, 31116 Republic of Korea; 2 Department of Nanobiomedical Science and BK21 Four NBM Global Research Center for Regenerative Medicine, Dankook University, Cheonan, 31116 Republic of Korea; 3 Mechanobiology Dental Medicine Research Center, Dankook University, Cheonan, 31116 Republic of Korea; 4 Department of Nanobiomedical Science and BK21 PLUS NBM Global Research Center for Regenerative Medicine, Dankook University, Cheonan, 31116 Republic of Korea; 5 Department of Biomaterials Science, College of Dentistry, Dankook University, Cheonan, 31116 Republic of Korea; 6 Department of Regenerative Dental Medicine, College of Dentistry, Dankook University, Cheonan, 31116 Republic of Korea

**Keywords:** Ultraviolet A, Oxidative stress, Human dermal fibroblasts, Hyaluronic acid, Polynucleotides, Dermal regeneration, Cell biology, Diseases, Medical research, Molecular biology

## Abstract

Ultraviolet A (UVA) radiation, a principal driver of skin photoaging, generates excessive reactive oxygen species (ROS) in dermal fibroblasts, causing oxidative stress, loss of viability, inflammatory signaling, and extracellular matrix (ECM) degradation. Hyaluronic acid (HA) and polynucleotides (PN) are clinically used dermal biomaterials; however, their protection against UVA injury remains insufficiently defined. We evaluated HA, PN, and their combination in human dermal fibroblasts (HDFs) subjected to photodamage. HDFs were pretreated with HA, PN, or both, irradiated with 20 J/cm^2^ UVA, and then maintained in treated media to mimic therapeutic recovery. UVA reduced viability and proliferation, downregulated ECM genes (*COL1A1*, *FN1*), and increased intracellular and mitochondrial ROS and proinflammatory cytokine gene (*TNF-α*). Monotherapy partially alleviated these changes. In contrast, combined HA + PN synergistically improved survival and proliferation, lowered ROS to near baseline, restored ECM transcription, and upregulated antioxidant enzymes (*GPX1*, S*OD2*). HA + PN also increased fibroblast invasion, indicating regenerative activity beyond cytoprotective effects. Under basal conditions, neither HA nor PN showed cytotoxicity or prooxidant effects, while modestly enhancing ECM transcription. These findings demonstrate that HA and PN act synergistically to counter UVA-induced oxidative stress and support dermal regeneration, highlighting a combinatorial bioactive strategy for photoaged skin.

**Supplementary Information:**

The online version contains supplementary material available at 10.1038/s41598-026-37730-5.

## Introduction

Human skin is constantly exposed to environmental stressors, among which ultraviolet A (UVA) radiation (320–400 nm) plays a predominant role in the process of cutaneous photoaging^1^. Unlike UVB, which primarily affects the epidermis, UVA penetrates deeply into the dermis and chronically perturbs the cellular microenvironment by generating reactive oxygen species (ROS)^2,3^. Human dermal fibroblasts (HDFs), the principal extracellular matrix (ECM)-producing cells in the dermis, are especially vulnerable to UVA-induced dysfunction^4–6^. Upon sustained UVA exposure, HDFs exhibit decreased viability and proliferation, disrupted cytoskeletal organization, and downregulated expression of key ECM components such as type I collagen (*COL1A1*) and fibronectin (*FN1*), leading to dermal atrophy, wrinkle formation, and impaired regenerative capacity^7,8^.

ROS generated by UVA irradiation cause cumulative damage to mitochondrial integrity, nuclear DNA, and cellular proteins, triggering a cascade of oxidative and inflammatory responses that impair dermal homeostasis^9^. At the molecular level, oxidative stress activates mitogen-activated protein kinase (MAPK) and nuclear factor kappa-light-chain-enhancer of activated B cells (NF-κB) signaling, which in turn upregulate matrix metalloproteinases (MMPs) responsible for collagen breakdown while downregulating ECM structural genes such as *COL1A1* and *FN1*^7,8^. In parallel, mitochondrial ROS accumulation disrupts antioxidant defenses, suppressing enzymes such as glutathione peroxidase (GPX1) and superoxide dismutase (SOD2). This redox imbalance not only accelerates ECM degradation but also amplifies inflammatory signaling cascades^10,11^. UVA exposure has been reported to alter cytokine profiles, including increased expression of tumor necrosis factor-α (TNF-α), a pro-inflammatory mediator, and decreased levels of interleukin-13 (IL-13), an anti-inflammatory cytokine involved in tissue remodeling^12,13^. Together, these processes establish a pathological loop in which excessive ROS, impaired ECM synthesis, and dysregulated inflammatory responses collectively drive photoaging and hinder dermal regeneration.

Hyaluronic acid (HA) is a naturally occurring glycosaminoglycan abundant in the dermal matrix, renowned for its hydrating and viscoelastic properties^14^. Beyond its structural role, HA modulates cell signaling, migration, and wound healing, and it has been extensively investigated in dermatology and regenerative skin research^15,16^. Polynucleotides (PN), derived from highly purified DNA fragments, are emerging as bioactive molecules that enhance fibroblast proliferation, regulate inflammation, and exert antioxidant activity^17^. PN-based materials have shown potential in skin and musculoskeletal research^18^. Importantly, previous studies suggest that HA and PN may independently influence oxidative stress, ECM remodeling, and inflammatory pathways^19^. However, their combined application under UVA-induced oxidative stress has not been systematically characterized. It is plausible that HA enhances dermal hydration and structural support, while PN modulates gene expression and redox balance, together producing synergistic protective effects^19,20^. Mechanistically, HA interacts with fibroblast surface receptors CD44 and receptor for hyaluronan-mediated motility (RHAMM), activating downstream phosphoinositide 3-kinase/protein kinase B (PI3K/Akt) and ERK pathways that enhance cell survival, cytoskeletal organization, and ECM synthesis^21^. This signaling promotes collagen and fibronectin production while maintaining pericellular hydration and matrix stability. In parallel, PN act through the adenosine A₂A receptor, stimulating cAMP/PKA/Akt cascades that alleviate oxidative stress, support mitochondrial function^22^. The convergence of these pathways at the Akt signaling node provides a mechanistic basis for the observed synergistic reduction of ROS and restoration of ECM integrity with HA + PN co-treatment. From a biomaterial’s perspective, therapeutic approaches targeting UVA-induced photoaging must simultaneously address oxidative stress, support cellular recovery, and stimulate ECM remodeling^23^. Conventional antioxidants, while capable of scavenging ROS, often suffer from poor cellular uptake, limited stability, and lack of regenerative signaling. Therefore, the development of bioactive materials that can both reduce oxidative damage and promote tissue regeneration remains an important need in skin biology and regenerative biomaterial research^24^.

In this study, we optimized a UVA-induced dermal fibroblast damage model and evaluated the protective and restorative potential of HA, PN, and their combined application. We focused on their effects on ROS accumulation, ECM and antioxidant gene expression, and cytokine regulation. Our findings demonstrate that while HA and PN individually confer moderate protective effects, their combination exerts a synergistic impact, thereby supporting a dual-component bioactive strategy for mitigating UVA-induced cellular damage.

## Results

### Optimization of UVA dosage and basal cytotoxicity of HA and PN

**Fig. 1:**
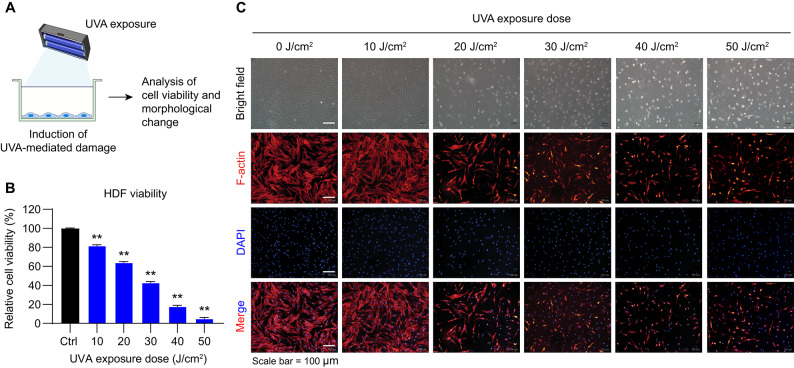
Effects of UVA irradiation on HDF viability and morphology. (**A**) Experimental scheme for UVA exposure and analysis. (**B**) Relative cell viability of HDFs exposed to increasing UVA doses (10–50 J/cm^2^), showing a dose-dependent decrease. (**C**) Representative images of F-actin (red) and DAPI (blue) staining demonstrate progressive cytoskeletal disruption and reduced cell density with higher UVA exposure. Scale bar = 100 µm. Values represent the mean ± SEM (n = 8). ^*^p < 0.05, ^**^p < 0.01 vs. control. Abbreviations: UVA, ultraviolet A; HDF, human dermal fibroblasts.

**Fig. 2:**
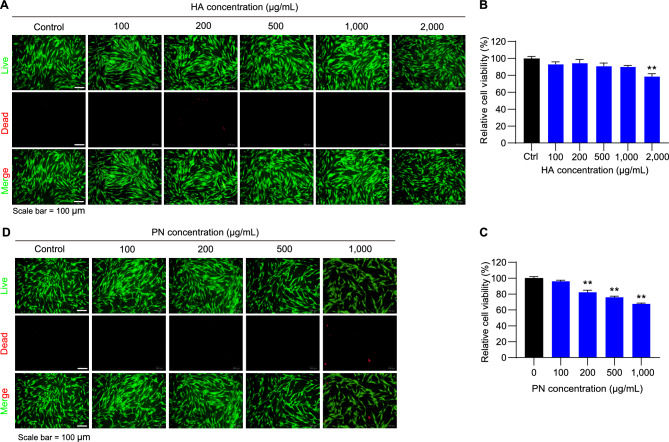
Effects of HA and PN on HDF viability. (**A**) Live/dead staining of HDFs treated with different concentrations of HA (100–2000 µg/mL). (**B**) Quantified viability showing significant reduction only at 2000 µg/mL HA. (**C**) Relative viability of HDFs treated with PN (100–1000 µg/mL). (**D**) Live/dead staining showing cytotoxicity at ≥ 200 µg/mL PN. Values represent the mean ± SEM (n = 7). ^*^p < 0.05, ^**^p < 0.01 vs. control. Abbreviations: HA, hyaluronic acid; PN, polynucleotide; HDF, human dermal fibroblasts.

To determine the appropriate UVA dose for establishing a dermal photodamage model, HDFs were exposed to increasing doses of UVA irradiation (0–50 J/cm^2^; Fig. 1A). Cell viability assays revealed a clear dose-dependent decline, with viability significantly reduced at 20 J/cm^2^ and dropping below 20% at 50 J/cm^2^ (Fig. 1B). Consistent with these results, morphological analysis showed progressive cytoskeletal disruption and reduced cell density with increasing UVA exposure, as visualized by F-actin and DAPI staining (Fig. 1C). Based on these findings, 20 J/cm^2^ was selected as the optimal dose for subsequent mechanistic and therapeutic evaluations, as it induced consistent cellular stress while maintaining sufficient survival for recovery analysis. Having established the challenge dose, we next assessed the basal cytotoxicity of HA and PN in the absence of UVA irradiation. Live/dead staining showed that low concentrations of HA (≤ 1000 μg/mL) and PN (≤ 100 μg/mL) did not alter HDF morphology or viability (Fig. 2A, C). Quantitative analysis of cell viability further confirmed that these concentrations had negligible effects. In contrast, higher concentrations of HA (≥ 2000 μg/mL) and PN (≥ 200 μg/mL) markedly reduced cell viability and led to a higher proportion of dead cells (Fig. 2B, D). These results indicate that both HA and PN are well tolerated at low to moderate doses but can compromise HDF survival when applied at elevated concentrations, independent of UVA exposure.

### Optimization of UVA exposure conditions and protective effects of HA and PN

**Fig. 3:**
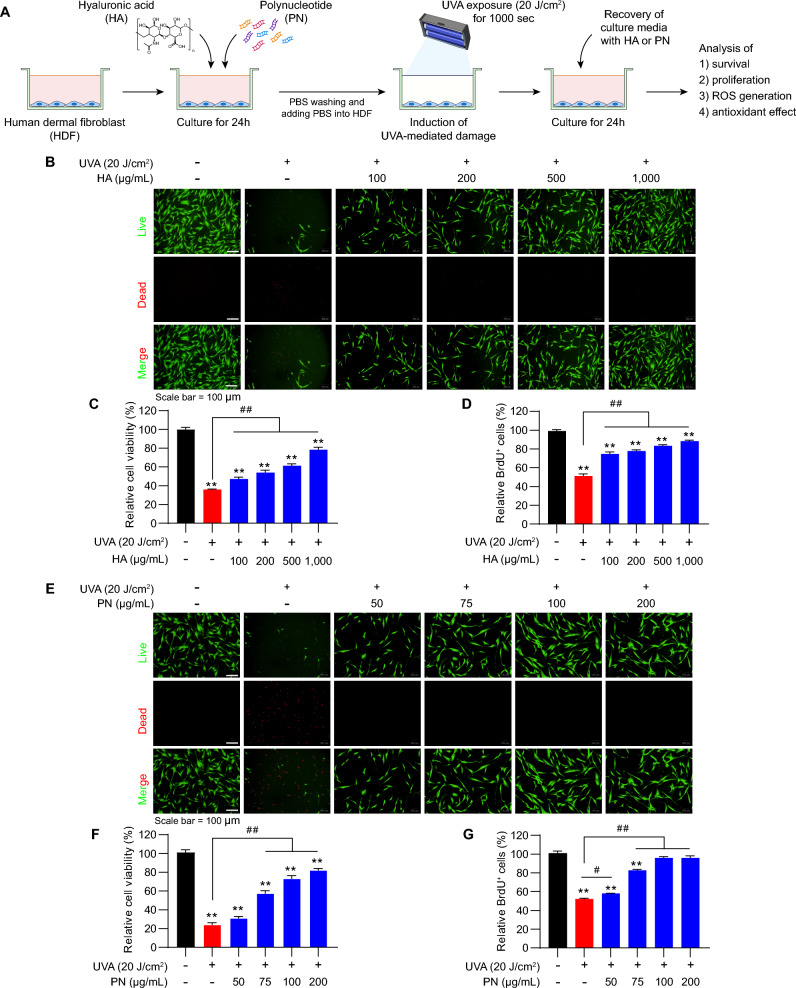
Protective effects of HA and PN in the pre-treatment–UVA irradiation–post-treatment model. (**A**) Experimental scheme illustrating the sequence of pre-treatment with HA or PN, UVA irradiation at 20 J/cm^2^, and subsequent post-treatment. (**B**, **E**) Live/dead staining showing improved survival with HA (100–1000 µg/mL) or PN (50–200 µg/mL). (**C**, **F**) Relative cell viability and (**D**, **G**) BrdU proliferation data confirming dose-dependent protection. Values represent the mean ± SEM (n = 5). ^*^p < 0.05, ^**^p < 0.01 vs. control; ^#^*p* < 0.05, ^##^*p* < 0.01 between indicated groups. Abbreviations: HA, hyaluronic acid; PN, polynucleotide; UVA, ultraviolet A.

The protective effect of HA and PN pre-treatment was assessed under different UVA irradiation conditions. HDFs were pre-treated for 24 h, exposed to 10 J/cm^2^ UVA for 500 s, and then maintained in HA- or PN-containing media (Supplementary Fig. S1A). This condition resulted in dose-dependent improvements in survival and proliferation, particularly at 500–1000 μg/mL HA and 50–200 μg/mL PN (Supplementary Fig. S1B–G). When recovery was conducted in treatment-free media after exposure to either 20 J/cm^2^ for 1000 s or 10 J/cm^2^ for 500 s, pre-treatment alone conferred only partial protection, as evidenced by modest increases in viability and proliferation compared with irradiated controls, but with substantially weaker effects than those observed under continuous treatment (Supplementary Figs. S2B-G, S3B-F). These results indicate that while HA or PN pre-treatment can prime fibroblasts against UVA-induced stress, the protective effect is limited in the absence of post-treatment support. Building on these findings, we established an optimized scheme in which HDFs were pre-treated with HA or PN, exposed to 20 J/cm^2^ UVA for 1000 s, and subsequently maintained in media containing the same treatment (Fig. 3A). Under this regimen, HA markedly improved cell survival and preserved morphology, with the strongest effects observed at 500–1000 μg/mL (Fig. 3B). Quantitative assays confirmed significant increases in both viability and proliferation compared with UVA-only controls (Fig. 3C, D). PN treatment showed a similar effect, with 100 μg/mL effectively restoring cell survival and reducing cytotoxicity (Fig. 3E). Quantitative analysis further confirmed significant improvements in both survival and proliferative activity relative to untreated irradiated groups (Fig. 3F, G). Collectively, these findings demonstrate that combining pre- and post-treatment with HA or PN provides stronger cytoprotection than pre-treatment alone, underscoring their therapeutic potential in mitigating UVA-induced photodamage.

### Synergistic effects of co-treatment with HA and PN on fibroblast recovery and invasion

**Fig. 4:**
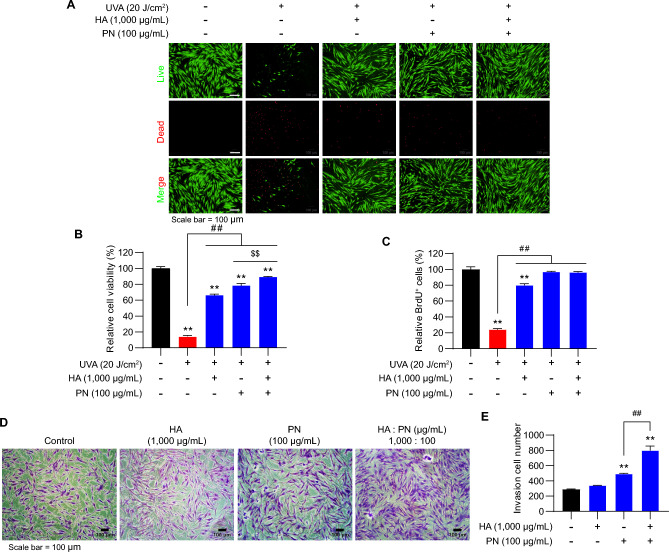
Synergistic protective effects of HA and PN against UVA-induced cytotoxicity. (**A**) Live/dead staining of HDFs exposed to UVA (20 J/cm^2^) with or without HA (1000 µg/mL), PN (100 µg/mL), or both. (**B**) Relative cell viability and (**C**) BrdU incorporation showing additive and synergistic improvements with the combination. (**D**) Representative morphology and invasion assay images. (**E**) Quantified invasion demonstrates a significant synergistic increase with co-treatment. Data are shown as mean ± SEM. (A–C, n = 10; D-E, n = 3). ^*^p < 0.05, ^**^p < 0.01 vs. control; ^##^*p* < 0.01, ^$$^*p* < 0.01 between indicated groups. Abbreviations: HA, hyaluronic acid; PN, polynucleotide; UVA, ultraviolet A; HDF, human dermal fibroblasts.

To determine whether HA and PN exert cooperative effects in protecting fibroblasts from UVA-induced stress, HDFs were exposed to 20 J/cm^2^ UVA for 1000 s and then cultured for 24 h in media containing HA (1,000 μg/mL), PN (100 μg/mL), or their combination. Co-treatment with HA and PN markedly increased the proportion of viable cells and reduced dead cell staining compared with either single treatment (Fig. 4A). Quantitative analyses showed that the combined treatment significantly enhanced cell viability and proliferation, surpassing the outcomes observed with HA or PN alone (Fig. 4B, C). In addition to survival and proliferation, we investigated whether HA and PN influence fibroblast motility under non-irradiated conditions. Transwell invasion assays showed that both HA and PN individually promoted fibroblast invasion compared with untreated controls. Representative crystal violet staining images revealed a greater number of invaded cells in the presence of HA or PN, with the highest invasion observed under combined treatment (Fig. 4D). Quantitative analysis confirmed that co-treatment significantly increased invasion cell numbers compared with either agent alone, indicating a synergistic effect on fibroblast migration (Fig. 4E). Together, these findings demonstrate that HA and PN synergistically protect fibroblasts from UVA-induced damage and promote functional properties related to dermal regeneration.

### Co-treatment with HA and PN suppresses UVA-induced oxidative stress and supports dermal homeostasis

**Fig. 5:**
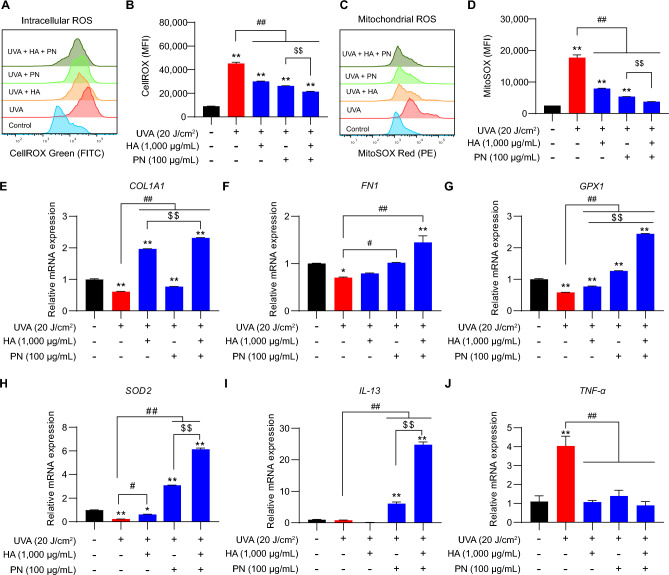
Effects of HA and PN on UVA-induced oxidative stress and recovery in HDFs. (**A–D**) Intracellular and mitochondrial ROS levels measured by flow cytometry. (**E–H**) Relative expression of *COL1A1*, *FN1*, *GPX1*, and *SOD2* in UVA-exposed HDFs. (**I–J**) Cytokine mRNA expression profiles showing UVA-induced *TNF-α* upregulation and *IL-13* downregulation, both normalized by treatments. Data are shown as mean ± SEM. (A–D, n = 5; E-J, n = 3). ^*^p < 0.05, ^**^p < 0.01 vs. control; ^#^*p* < 0.05, ^##^*p* < 0.01; ^$$^*p* < 0.01 between indicated groups. Abbreviations: HA, hyaluronic acid; PN, polynucleotide; UVA, ultraviolet A; HDF, human dermal fibroblasts; ROS, reactive oxygen species; COL1A1, collagen type I alpha 1 chain; FN1, fibronectin 1; GPX1, glutathione peroxidase 1; SOD2, superoxide dismutase 2; TNF-α, tumor necrosis factor-alpha; IL-13, interleukin-13.

To examine the effects of HA and PN on oxidative stress and related pathways, intracellular ROS was first assessed using CellROX Green after UVA irradiation. UVA significantly elevated cytoplasmic ROS compared with untreated controls (Fig. 5A), and quantitative analysis confirmed a strong increase in mean fluorescence intensity (Fig. 5B). HA or PN treatment reduced ROS levels, while the combination restored intracellular ROS to near-control values. Under non-irradiated conditions, cytoplasmic ROS remained stable across all groups, showing that HA and PN have no basal pro-oxidant effect (Supplementary Fig. S4A, B). Mitochondrial superoxide was then measured using MitoSOX Red. UVA strongly elevated mitochondrial ROS (Fig. 5C). Quantitative analysis confirmed that HA or PN partially reduced these levels, with the most effective suppression observed under co-treatment (Fig. 5D). Consistent with cytoplasmic ROS, mitochondrial ROS also remained unchanged under non-irradiated conditions regardless of treatment (Supplementary Fig. S4C and D). At the transcriptional level, UVA exposure significantly suppressed ECM-related genes, as evidenced by reduced expression of *COL1A1* and *FN1* (Fig. 5E, F). Both HA and PN partially restored the expression of these genes, while the combination achieved near-complete recovery. UVA also downregulated the antioxidant genes *GPX1* and *SOD2*, and their expression was most effectively restored by co-treatment with HA and PN (Fig. 5G, H). In non-irradiated cells, HA and PN modestly upregulated *COL1A1, FN1, GPX1*, and *SOD2*, indicating a basal pro-regenerative and antioxidant-supportive role (Supplementary Fig. S4E–H). Cytokine-related gene expressions showed that UVA irradiation markedly increased the pro-inflammatory cytokine *TNF-α* mRNA expression, while suppressing the anti-inflammatory cytokine *IL-13* mRNA expression (Fig. 5I, J). Treatment with HA or PN partially reversed these changes, whereas the combination most effectively reduced *TNF-α* mRNA levels and restored *IL-13* mRNA expression. Under non-irradiated conditions, cytokine mRNA levels remained stable, supporting the immunomodulatory safety of HA and PN (Supplementary Fig. S4I and J). In addition, Bliss independence analysis demonstrated synergistic effect of HA + PN on cell migration and molecular recovery (Supplementary Fig. S5A-F). To further examine the relationship between oxidative stress attenuation and molecular recovery, we compared the extent of ROS suppression with the magnitude of transcriptional restoration. Groups exhibiting stronger reductions in intracellular and mitochondrial ROS (Fig. 5B, D) also showed greater recovery of ECM-related genes (*COL1A1*, *FN1*) and antioxidant genes (*GPX1*, *SOD2*) (Fig. 5E–H). This inverse relationship suggests that oxidative stress directly constrains ECM and antioxidant gene transcription, and that its alleviation by HA + PN enables coordinated molecular and functional recovery. Consistent with this relationship, the co-treated group, which exhibited near-baseline ROS levels, also showed the highest cell viability and invasion capacity (Fig. 4C, 6B). To evaluate antioxidant efficacy, vitamin C (ascorbic acid, 150 µM) was included as a positive control. Vitamin C effectively mitigated UVA-induced cytotoxicity and reduced intracellular ROS to a level comparable to that of HA + PN under 10 J/cm^2^ UVA exposure, but its protective effect was notably weaker at 20 J/cm^2^ (Supplementary Fig. S6), supporting the superior redox-regenerative performance of the HA + PN combination. Collectively, these findings indicate that HA and PN synergistically reduce UVA-induced oxidative stress, restore ECM and antioxidant gene expression, and normalize cytokine homeostasis.

**Fig. 6:**
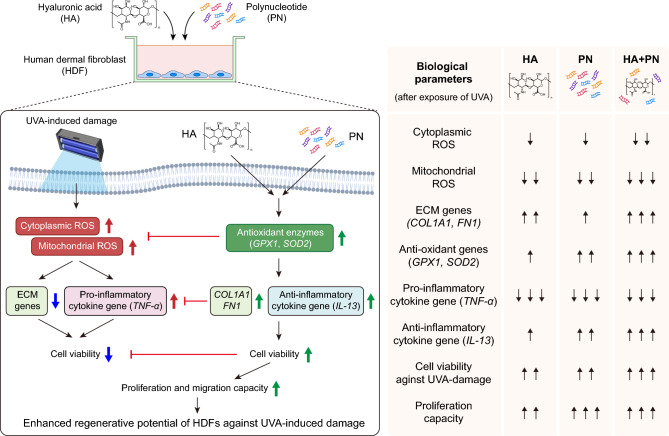
Schematic illustration of the synergistic effect of HA and PN on UVA-induced damage in HDFs. Under UVA exposure, cytoplasmic and mitochondrial ROS accumulate, which decreases ECM gene expression and increases pro-inflammatory cytokine gene expression (e.g., *TNF-α*), ultimately reducing HDF viability. Co-treatment with HA and PN enhances antioxidant enzyme genes (e.g., *GPX1* and *SOD2*), thereby lowering UVA-induced ROS. Consequently, ECM genes (e.g., *COL1A1* and *FN1*) and the anti-inflammatory cytokine gene (e.g., *IL-13*) are upregulated, supporting cell survival under UVA stress. These synergistic actions also promote HDF proliferation and migration, thereby increasing the regenerative potential of HDFs in the context of UVA-induced damage. The right panel compares the beneficial effects of HA + PN co-treatment with those of HA or PN alone under UVA exposure. Abbreviations: HA, hyaluronic acid; PN, polynucleotide; UVA, ultraviolet A; HDF, human dermal fibroblasts; ROS, reactive oxygen species; ECM, extracellular matrix; TNF-α, tumor necrosis factor-alpha; GPX1, glutathione peroxidase 1; SOD2, superoxide dismutase 2; COL1A1, collagen type I alpha 1 chain; FN1, fibronectin 1; IL-13, interleukin-13.

## Discussion

Our study first established a reproducible in vitro model of UVA-induced photodamage, where exposure at 20 J/cm^2^ consistently induced oxidative stress, cytoskeletal disorganization, and reduced viability in dermal fibroblasts without causing complete lethality. This finding is consistent with previous reports demonstrating that UVA penetrates into the dermis, elevates intracellular ROS, and downregulates ECM synthesis^25–28^. The choice of 20 J/cm^2^ as a challenge dose is in line with thresholds reported for dermal photoaging studies, ensuring a balance between measurable cytotoxicity and recovery potential. By validating this model, we provide a robust platform to test therapeutic interventions that target both acute oxidative stress and subsequent regenerative capacity.

We observed that HA and PN, when applied individually, protected fibroblasts against UVA-induced damage in a concentration-dependent manner. Moderate concentrations of HA (≤ 1000 μg/mL) and PN (≤ 100 μg/mL) improved survival and proliferation, whereas supra-physiological doses were detrimental under non-stressed conditions. This biphasic, hormetic response aligns with prior studies of biologically active polymers and nucleotides, where excessive concentrations disrupted osmotic balance, receptor signaling, or nucleotide metabolism^29–31^. Previous research has shown that HA promotes ECM stability and fibroblast migration through CD44- and RHAMM pathways^32,33^, while PN stimulates proliferation and reduces inflammation through adenosine receptor activation^22,34,35^. In addition, HA has been reported to facilitate fibroblast recovery by stabilizing pericellular hydration and maintaining matrix architecture, whereas PN may promote DNA repair and metabolic reactivation through adenosine receptor–dependent signaling^22,36,37^. The synergistic protection observed with HA + PN appears to arise from the integration of extracellular matrix stabilization and intracellular redox regulation. Rather than acting through a single pathway, the two components engage complementary mechanisms that collectively maintain cellular homeostasis under oxidative stress. HA primarily supports the extracellular environment by preserving hydration and structural integrity of the matrix, thereby maintaining fibroblast morphology and signaling responsiveness^32,33,36^. PN, in turn, modulates intracellular redox balance and antioxidant signaling through adenosine receptor–mediated pathways^22,35,37^. The combination of these extracellular and intracellular actions likely amplifies fibroblast recovery by linking matrix integrity with metabolic and redox balance. Such cross-compartment cooperation between matrix-mediated signaling and nucleotide-derived metabolic activation provides a plausible explanation for the robust restoration of antioxidant capacity, ECM gene expression, and cytokine homeostasis observed in this study. Our findings confirm these individual benefits but also underscore the importance of dose optimization, as therapeutic effects under stress may not translate directly to non-stressed tissues.

The most significant finding of this work is the synergistic effect observed when HA and PN were applied together. Co-treatment not only improved survival and proliferation beyond single-agent effects but also enhanced fibroblast motility, as evidenced by transwell invasion assays. HA and PN suppressed both cytoplasmic and mitochondrial ROS, a dual-compartment control likely critical for long-term photoprotection. Previous studies have shown that excessive mitochondrial ROS can drive fibroblast senescence through activation of the p38 MAPK and NF-κB pathways, thereby perpetuating chronic inflammation and ECM degradation^38–40^. At the transcriptional level, UVA-induced suppression of *COL1A1* and *FN1* was nearly reversed by co-treatment, consistent with reports of HA activating PI3K/Akt signaling to enhance ECM production and PN modulating anabolic gene expression through purinergic pathways^32–35^. In parallel, antioxidant genes (*GPX1*, *SOD2*) were restored, while cytokine responses were normalized: *TNF-α* mRNA expression was suppressed and *IL-13* mRNA expression was restored. This normalization of cytokine signaling is likely to attenuate downstream activation of MMPs, thereby reducing collagen breakdown and reinforcing ECM preservation^41,42^.

The dual protective and regenerative actions of HA and PN may have important implications for regenerative skin biology. Interventions that both suppress oxidative damage and promote dermal regeneration remain of interest in the context of photoaging research. Our findings suggest that HA + PN could represent a bioactive strategy relevant to conditions involving controlled dermal injury and subsequent repair. Moreover, the absence of pro-oxidant activity under non-stressed conditions suggests safety for further exploration as a biomaterial-based approach in skin research. Compared with conventional antioxidants, which often lack regenerative signaling^43^, the HA + PN strategy provides both structural and biochemical support, positioning it as a promising dual-component bioactive therapy (Fig. 6). From a translational and clinical perspective, the synergistic antioxidant and regenerative effects demonstrated in this study hold strong potential for application in aesthetic and regenerative dermatology. Recent reviews have emphasized that the future of anti-photoaging therapies lies in bioactive antioxidant biomaterials capable of simultaneously restoring extracellular matrix integrity and modulating redox homeostasis^44–46^. Such approaches are increasingly supported by advanced material platforms, particularly hydrogels and injectable composite systems that combine structural support with controlled antioxidant or bioactive release^47,48^. The HA + PN combination evaluated here aligns closely with these translational trends. HA provides mechanical cushioning, hydration, and viscoelasticity, while PN contributes nucleotide-derived bioactivity that enhances fibroblast regeneration, antioxidant defense, and ECM synthesis. These dual mechanisms parallel the design philosophy of emerging HA-based dermal fillers and skin booster formulations, which aim to integrate hydration, cellular repair, and oxidative protection within a single treatment platform. Indeed, clinical meta-analyses of HA injectables for skin aging^49^ and recent reports on composite filler systems^50^ highlight similar goals of achieving both structural and biochemical rejuvenation. Therefore, our findings provide a mechanistic foundation supporting the development of next-generation bioactive dermal fillers and hydrogel formulations that combine HA’s physical functions with PN’s antioxidant and regenerative signaling. Such dual-component strategies may represent a promising path toward clinically relevant anti-photoaging and skin-rejuvenation therapies that bridge the gap between biomaterial engineering and functional tissue restoration.

Despite these encouraging results, several limitations should be acknowledged. First, this study utilized a two-dimensional fibroblast culture model, which does not capture the complexity of skin architecture involving keratinocytes, immune cells, and vascular networks^51^. Second, the investigation was restricted to short-term responses (24–48 h) under acute UVA exposure. Future studies should extend these findings to chronic or repeated UVA irradiation models to evaluate long-term processes such as fibroblast senescence, ECM remodeling, and sustained inflammatory changes. Third, while we implicated pathways such as CD44/HA and adenosine receptor signaling, the precise mechanisms of synergy were not dissected using targeted inhibitors or genetic tools. Moreover, this work focused primarily on transcriptional responses; although *COL1A1* and *FN1* mRNA recovery suggests matrix restoration, protein-level confirmation is still required to verify actual ECM deposition and remodeling. Future work will therefore include western blotting and ELISA analyses for type I procollagen α1 and fibronectin, complemented by immunofluorescence imaging of ECM organization. Finally, the direct supplementation of HA and PN in culture media bypasses important translational challenges, including formulation stability, dermal penetration, and delivery efficiency^52^. Follow-up investigations using fibroblast–keratinocyte co-cultures and advanced 3D skin equivalents, together with optimized hydrogel or nanoparticle delivery systems, will be essential to confirm the mechanistic and therapeutic relevance of HA + PN in vivo. Similar integrative multicellular and protein-level approaches have been emphasized in recent reviews on bioactive antioxidants^53,54^.

## Methods

### Cell culture

PrimaPure™ HDFs were purchased from Genlantis (San Diego, CA, USA). Cells were cultured in high-glucose Dulbecco’s Modified Eagle Medium (DMEM; Thermo Fisher Scientific, Waltham, MA, USA) supplemented with 10% fetal bovine serum (FBS; Thermo Fisher Scientific) and 1% penicillin–streptomycin (PS; Gibco, USA). Cultures were maintained in a humidified incubator at 37 °C with 5% CO₂. Cells were passaged using 0.05% trypsin–EDTA (Thermo Fisher Scientific) upon reaching 80–90% confluency. HDFs between passages 6 and 10 were used for all experiments.

### Materials

Hyaluronic acid (HA; molecular weight: approximately 920–1080 kDa) and polynucleotides (PN; molecular weight: approximately 1900–2500 kDa) were supplied by GENOSS Co., Ltd. (Suwon, Korea). The HA used in this study was a commercially manufactured product, whereas PN was derived from a natural source and extracted from the testis of salmon (*Oncorhynchus keta*). Both materials were provided in sterile, lyophilized form and freshly reconstituted in culture medium prior to use.

### Pre-treatment—UVA irradiation—post-treatment

HDFs were seeded in 96-well plates (4 × 10^3^ cells/well), 30 mm cell culture dishes (1 × 10^5^ cells/dish), or 60 mm cell culture dishes (1 × 10^6^ cells/dish) depending on the assay. After 24 h, cells were treated with HA, PN, or a combination of both by supplementing the DMEM medium with treatment solutions at 10% of the total volume. Control groups received DMEM only. Treatments were maintained for 24 h. Before UVA exposure, the medium was removed and replaced with a thin layer of PBS. Cells were then irradiated using a Lumen Dynamics S1500A system (365 nm, 20 mW/cm^2^, Lumen Dynamics Group Inc., Mississauga, Canada) under optimized conditions, as described in previous studies employing UVA-induced photoaging models in HDFs^5,8^. Following irradiation, PBS was replaced with fresh DMEM containing the same treatment (10% v/v), and cells were incubated for the indicated durations prior to analysis. For positive control, cells were treated with 150 µM ascorbic acid (vitamin C; Sigma-Aldrich, St. Louis, MO, USA) for 24 h prior to UVA exposure and maintained in vitamin C–containing medium during the post-treatment recovery phase. The UVA irradiation and analytical procedures were identical to those described above. The concentration of 150 µM was selected based on previous reports showing effective antioxidant protection of human dermal fibroblasts under comparable UVA-induced oxidative stress conditions^6,55^.

### Visualization of UVA-induced morphological alterations

After UVA exposure, HDFs were fixed with 4% paraformaldehyde for 15 min and permeabilized with 0.1% Triton X-100 in PBS for 10 min. Cells were then stained with Alexa Fluor™ 546 Phalloidin (Thermo Fisher Scientific) at a dilution of 1:400 for 30 min in the dark. Nuclei were counterstained with 4′,6-diamidino-2-phenylindole (DAPI; Sigma-Aldrich) at a final concentration of 1 µg/mL for 5 min. After PBS washes, samples were imaged using an IX73 inverted microscope (Olympus, Tokyo, Japan) to assess cell morphology.

### In vitro cytotoxicity assay

Cell viability was evaluated using a Cell Counting Kit-8 (CCK-8; Dojindo Laboratories, Kumamoto, Japan) according to the manufacturer’s instructions. Following the pre-treatment—UVA irradiation—post-treatment procedures described in the preceding section, 10 µL of CCK-8 reagent was added to each well containing 100 µL of culture medium, followed by incubation for 2 h at 37 °C. Plates were then measured at 450 nm using the Varioskan LUX Multimode microplate reader (Thermo Fisher Scientific). Cell viability was normalized to the untreated control group. For live/dead analysis, cells were stained with 2 µM Calcein AM and 4 µM Ethidium homodimer-1 (EthD-1; Thermo Fisher Scientific) in PBS for 30 min at room temperature. Fluorescence images were acquired using an IX73 inverted microscope (Olympus).

### 5-Bromo-2-deoxyuridine (BrdU) cell proliferation assay

Cell proliferation was evaluated using the BrdU Cell Proliferation Assay Kit (Cell Signaling Technology, Danvers, MA, USA), following the manufacturer’s instructions. After pre-treatment and UVA irradiation as described above, HDFs were incubated with BrdU for 24 h in DMEM containing the respective treatment. Cells were then fixed, immunostained with anti-BrdU antibody and horseradish peroxidase-conjugated secondary antibody, and absorbance was measured at 450 nm using the Varioskan LUX Multimode microplate reader (Thermo Fisher Scientific).

### Cell invasion assay

Transwell inserts with 8.0 μm pore polycarbonate membranes (Corning Inc., Corning, NY, USA) were pre-coated with 0.1% gelatin. HDFs (4 × 10^5^ cells/chamber) were seeded into the upper chambers in DMEM media and incubated for attachment. After 2–4 h, the medium was gently replaced with DMEM media containing HA, PN, or their combination. The lower chambers were filled with DMEM supplemented with 10% FBS to serve as a chemoattractant. After 24 h of incubation at 37 °C, non-invaded cells on the upper surface were removed with a cotton swab. Invaded cells on the lower surface were fixed with 4% paraformaldehyde, stained with 0.5% crystal violet, and rinsed with distilled water. Membranes were imaged using an IX73 inverted microscope (Olympus), and the invaded area was quantified using ImageJ (NIH, Bethesda, MD, USA) by color thresholding and particle analysis.

### Measurement of intracellular reactive oxygen species (ROS)

Intracellular reactive oxygen species (ROS) levels were assessed using CellROX™ Green and MitoSOX™ Red reagents (Thermo Fisher Scientific), following the manufacturer’s protocols. CellROX™ Green was used to detect total intracellular ROS, while MitoSOX™ Red was used to specifically measure mitochondrial superoxide. After pre-treatment and UVA irradiation as described above, HDFs were incubated for the indicated durations in DMEM containing the respective treatment. Cells were then harvested, washed with Hank’s Balanced Salt Solution (HBSS), and stained with 5 μM CellROX™ Green for 30 min or 5 μM MitoSOX™ Red for 10 min at 37 °C in the dark. After staining, cells were washed, resuspended in PBS, and analyzed by flow cytometry using a BD FACSCanto II system (BD Biosciences, San Jose, CA, USA). Data were processed and quantified using FlowJo software (BD Biosciences).

### RNA extraction and cDNA synthesis

Total RNA was extracted using the Ribospin™ kit (GeneAll, Seoul, Korea). RNA concentration and purity were measured with a NanoDrop™ 2000 spectrophotometer (Thermo Fisher Scientific). cDNA was synthesized from 1 μg of total RNA using the iScript™ cDNA Synthesis Kit (Bio-Rad, Hercules, CA, USA), following the manufacturer’s instructions. The resulting cDNA was stored at − 20 °C until use.

### Quantitative real-time polymerase chain reaction assay (qPCR)

qPCR was performed using the QuantStudio™ 1 Real-Time PCR System (Applied Biosystems, Thermo Fisher Scientific) with SYBR® Green High ROX qPCR Master Mix (Enzynomics, Daejeon, Korea). Gene expression was analyzed using the comparative Ct method, with GAPDH as the reference gene. Supplementary Table 1 shows the primers used for qPCR. Melt curve analysis was performed to confirm specificity.

### Bliss independence analysis

The interaction between HA and PN was evaluated using the Bliss independence model. The predicted additive effect was calculated as *F*_pre_d = *F*_HA_ + *F*_PN_–*F*_HA_ × *F*_PN_, and compared with the observed combined response (*F*_oβs_). A Bliss index (*F*_oβs_/*F*_pre_d) > 1 indicates synergy^56,57^. Statistical comparisons were performed using one-sample *t*-tests against the theoretical additive value.

### Statistical analysis

Statistical analyses were performed using GraphPad Prism 8.4.3 software. Data is presented as the mean ± standard error of the mean (SEM). Each experiment was independently repeated multiple times, with at least three biological replicates per assay. The exact number of biological replicates (n) for each dataset is indicated in the corresponding figure legends. Comparisons among multiple groups were performed using one-way analysis of variance (ANOVA) followed by Dunnett’s post hoc test to compare treated groups with the control. Comparisons between two groups were assessed using an unpaired Student’s *t*-test. A *p*-value less than 0.05 was considered statistically significant (^*^p < 0.05; ^**^p < 0.01).

## Supplementary Information

Supplementary Information.

## Data Availability

The data of this study are available from the corresponding author upon reasonable request.
