# Correction: Synergistic protective and regenerative effects of hyaluronic acid and polynucleotides against UVA-induced oxidative stress in dermal fibroblasts

**DOI:** 10.1038/s41598-026-52942-5

**Published:** 2026-06-03

**Authors:** Trang Thanh Thien Tran, Soon Chul Heo, Jun Hee Lee, Hae-Won Kim

**Affiliations:** 1 Institute of Tissue Regeneration Engineering (ITREN), Dankook University, Cheonan, 31116 Republic of Korea; 2 Department of Nanobiomedical Science and BK21 Four NBM Global Research Center for Regenerative Medicine, Dankook University, Cheonan, 31116 Republic of Korea; 3 Mechanobiology Dental Medicine Research Center, Dankook University, Cheonan, 31116 Republic of Korea; 4 Department of Nanobiomedical Science and BK21 PLUS NBM Global Research Center for Regenerative Medicine, Dankook University, Cheonan, 31116 Republic of Korea; 5 Department of Biomaterials Science, College of Dentistry, Dankook University, Cheonan, 31116 Republic of Korea; 6 Department of Regenerative Dental Medicine, College of Dentistry, Dankook University, Cheonan, 31116 Republic of Korea

Correction to: *Scientific Reports* 10.1038/s41598-026-37730-5, published online 30 January 2026

The original version of this Article contained an error in Figure 1 panel (C), where the panel for the 50 J/cm^2^ group was inadvertently duplicated from the 40 J/cm^2^ group during the final figure assembly. The original Figure 1 and accompanying legend appear below.

**Fig. 1:**
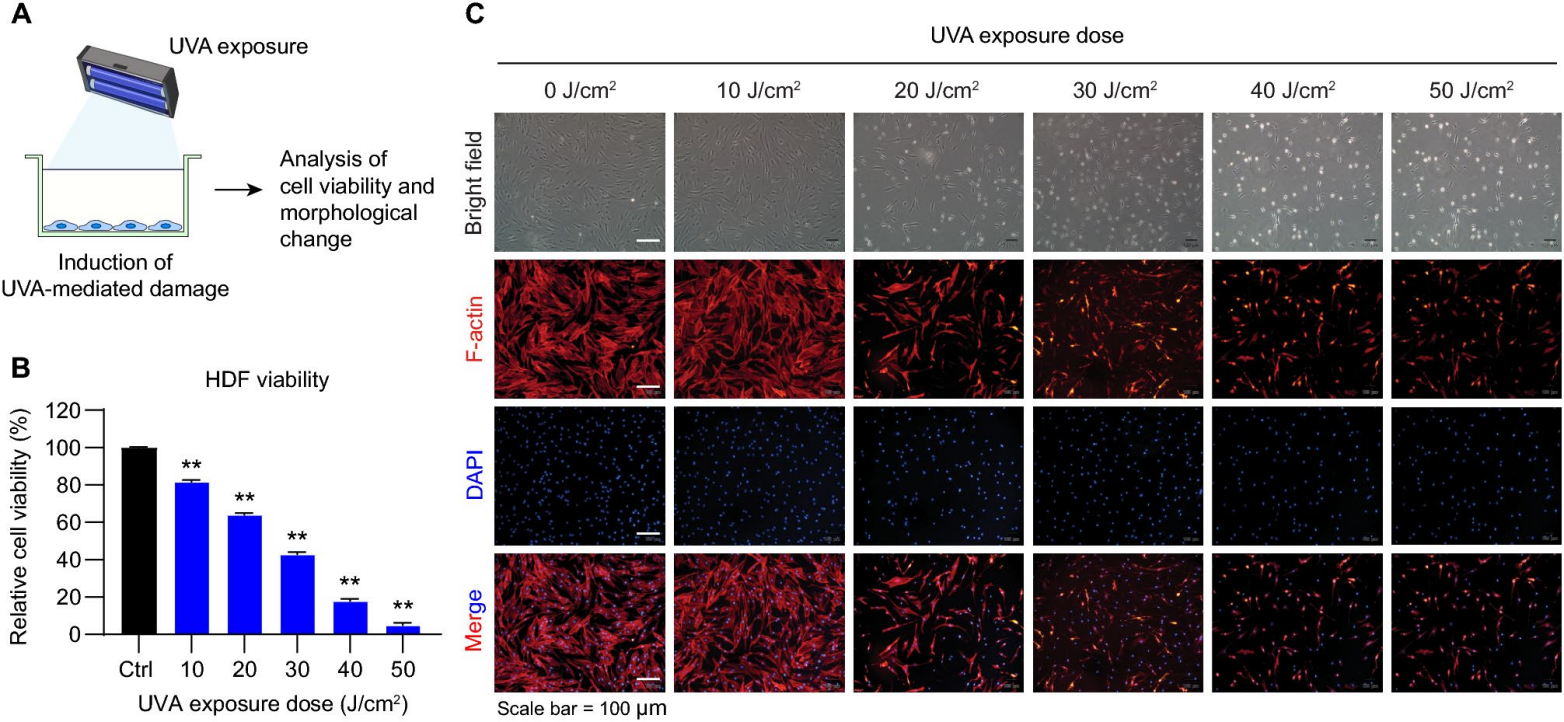
Effects of UVA irradiation on HDF viability and morphology. (**A**) Experimental scheme for UVA exposure and analysis. (**B**) Relative cell viability of HDFs exposed to increasing UVA doses (10–50 J/cm^2^), showing a dose-dependent decrease. (**C**) Representative images of F-actin (red) and DAPI (blue) staining demonstrate progressive cytoskeletal disruption and reduced cell density with higher UVA exposure. Scale bar = 100 µm. Values represent the mean ± SEM (n = 8). ^*^p < 0.05, ^**^p < 0.01 vs. control. Abbreviations: UVA, ultraviolet A; HDF, human dermal fibroblasts.

The original Article has been corrected.

