# Supplementary material for: Synergistic protective and regenerative effects of hyaluronic acid and polynucleotides against UVA-induced oxidative stress in dermal fibroblasts
Source: Sci Rep. 2026 Jan 30;16:6703. doi: 10.1038/s41598-026-37730-5 (PMC12913908; doi:10.1038/s41598-026-37730-5)
Supplement: Supplementary file 1 — Supplementary Information. [file 41598_2026_37730_MOESM1_ESM.docx]

**Supporting Information for: [**Synergistic protective and regenerative effects of hyaluronic acid and polynucleotides against UVA-induced oxidative stress in dermal fibroblasts**]**

**Supplementary Figures**


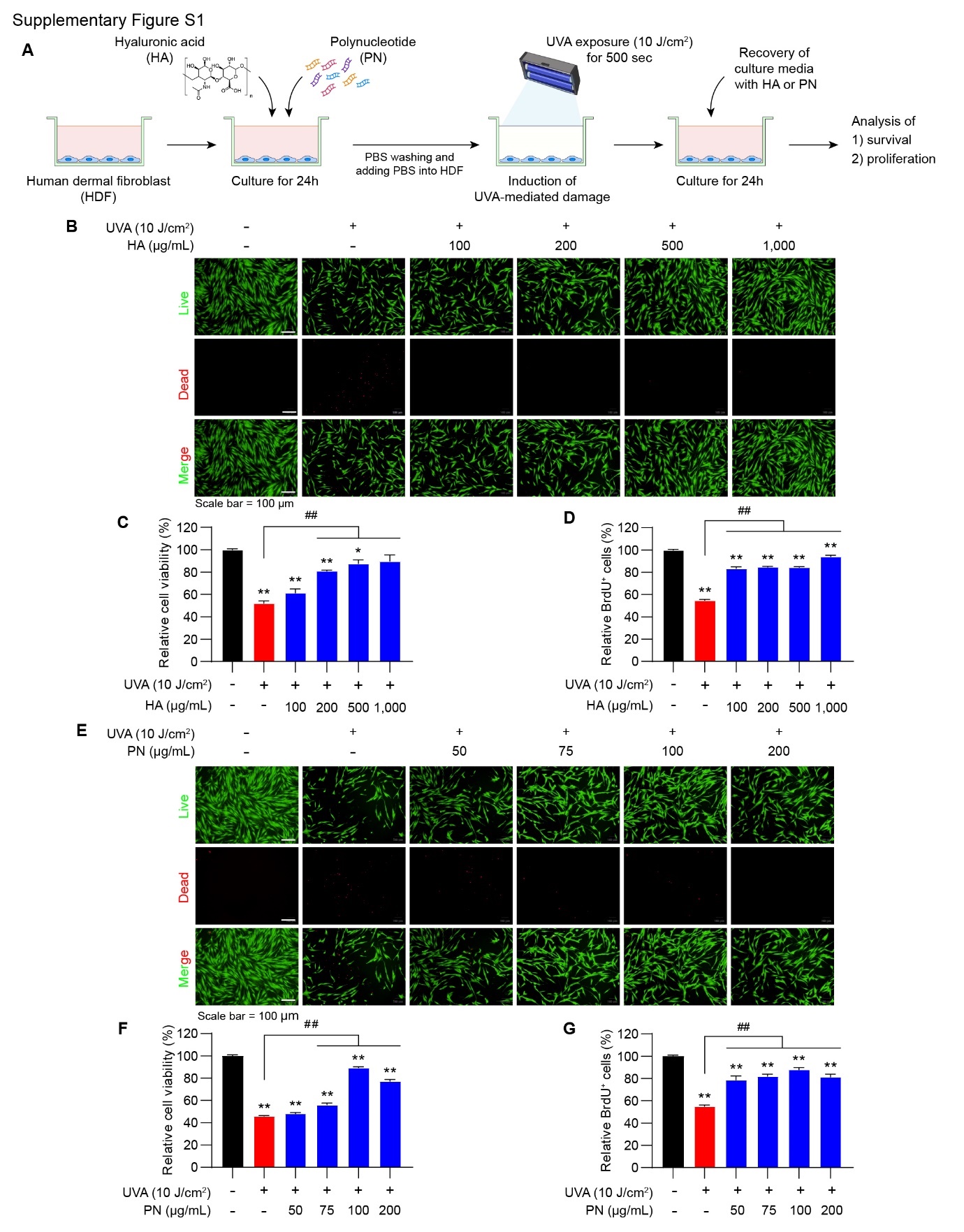


**Supplementary Figure S1. Protective effects of HA and PN against low-dose UVA-induced damage in HDFs.** (**A**) Schematic diagram of the experimental design (pre-treatment → UVA irradiation at 10 J/cm² → post-treatment). (**B, E**) Live/dead assay after treatment with increasing concentrations of HA (100–1,000 µg/mL) or PN (50–200 µg/mL). (**C, F**) Quantification of relative cell viability. (**D, G**) BrdU assay showing proliferative activity. Both HA and PN improved cell survival and proliferation in a dose-dependent manner after UVA exposure. Values represent the mean ± SEM (n = 5). ^**^*p* < 0.01 vs. control; ^##^*p* < 0.01 between indicated groups. Abbreviations: HA, hyaluronic acid; PN, polynucleotide; UVA, ultraviolet A; HDF, human dermal fibroblasts.

**
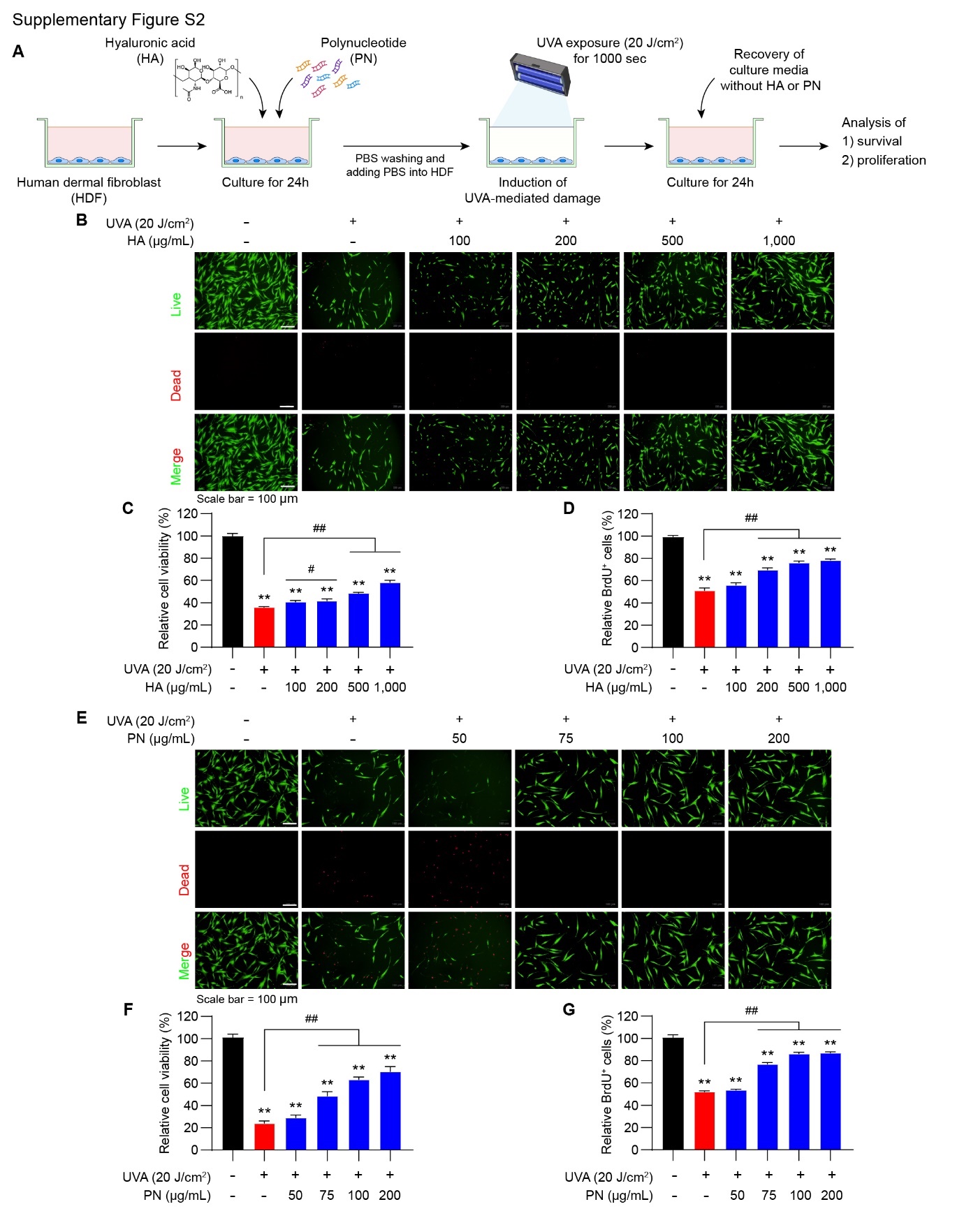
**

**Supplementary Figure S2. Pre-treatment effects of HA and PN on UVA-induced damage in HDFs.** (**A**) Schematic diagram of the experimental design (pre-treatment → UVA irradiation at 20 J/cm², no post-treatment). (**B, E**) Live/dead assay of HDFs pre-treated with HA (100–1,000 µg/mL) or PN (50–200 µg/mL) prior to UVA exposure. (**C, F**) Quantification of relative cell viability. (**D, G**) BrdU assay showing proliferative activity. Both HA and PN pre-treatment improved cell survival and proliferation in a dose-dependent manner. Values represent the mean ± SEM (n = 5). ^**^*p* < 0.01 vs. control; ^##^*p* < 0.01 between indicated groups. Abbreviations: HA, hyaluronic acid; PN, polynucleotide; UVA, ultraviolet A; HDF, human dermal fibroblasts.

**
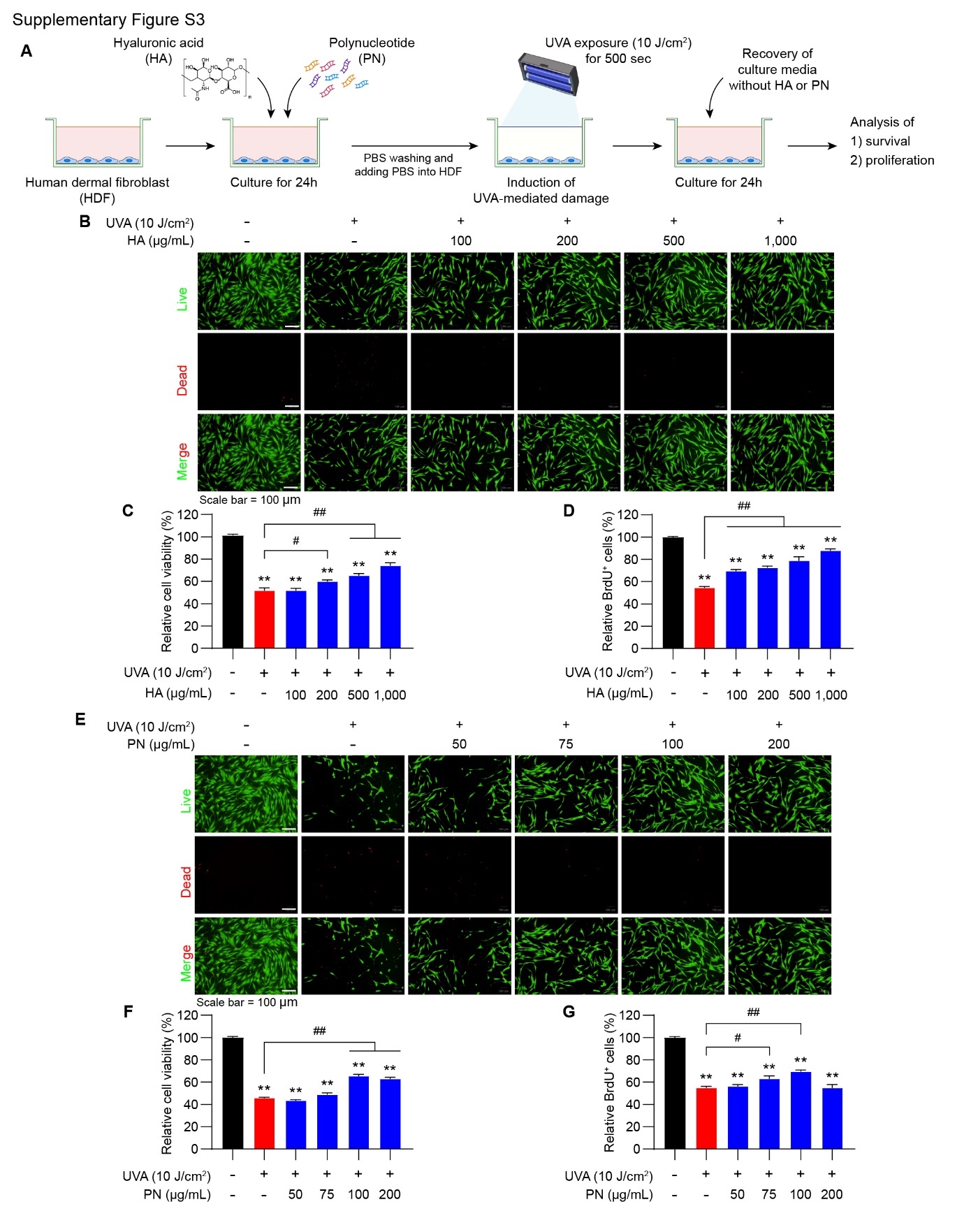
Supplementary Figure S3. Pre-treatment effects of HA and PN on UVA-induced damage (10 J/cm²) in HDFs.** (**A**) Schematic of the experimental design (pre-treatment → UVA irradiation at 10 J/cm², no post-treatment). (**B, D**) Live/dead assay of HDFs pre-treated with HA (100–1,000 µg/mL) or PN (50–200 µg/mL) prior to UVA exposure. (**C, E**) Quantification of relative cell viability. (**C, F**) BrdU assay showing proliferative activity. HA and PN pre-treatment preserved cell survival and proliferation against UVA 10 J/cm² in a concentration-dependent manner. Values represent the mean ± SEM (n = 5). ^**^*p* < 0.01 vs. control; ^##^*p* < 0.01 between indicated groups. Abbreviations: HA, hyaluronic acid; PN, polynucleotide; UVA, ultraviolet A; HDF, human dermal fibroblasts.

**
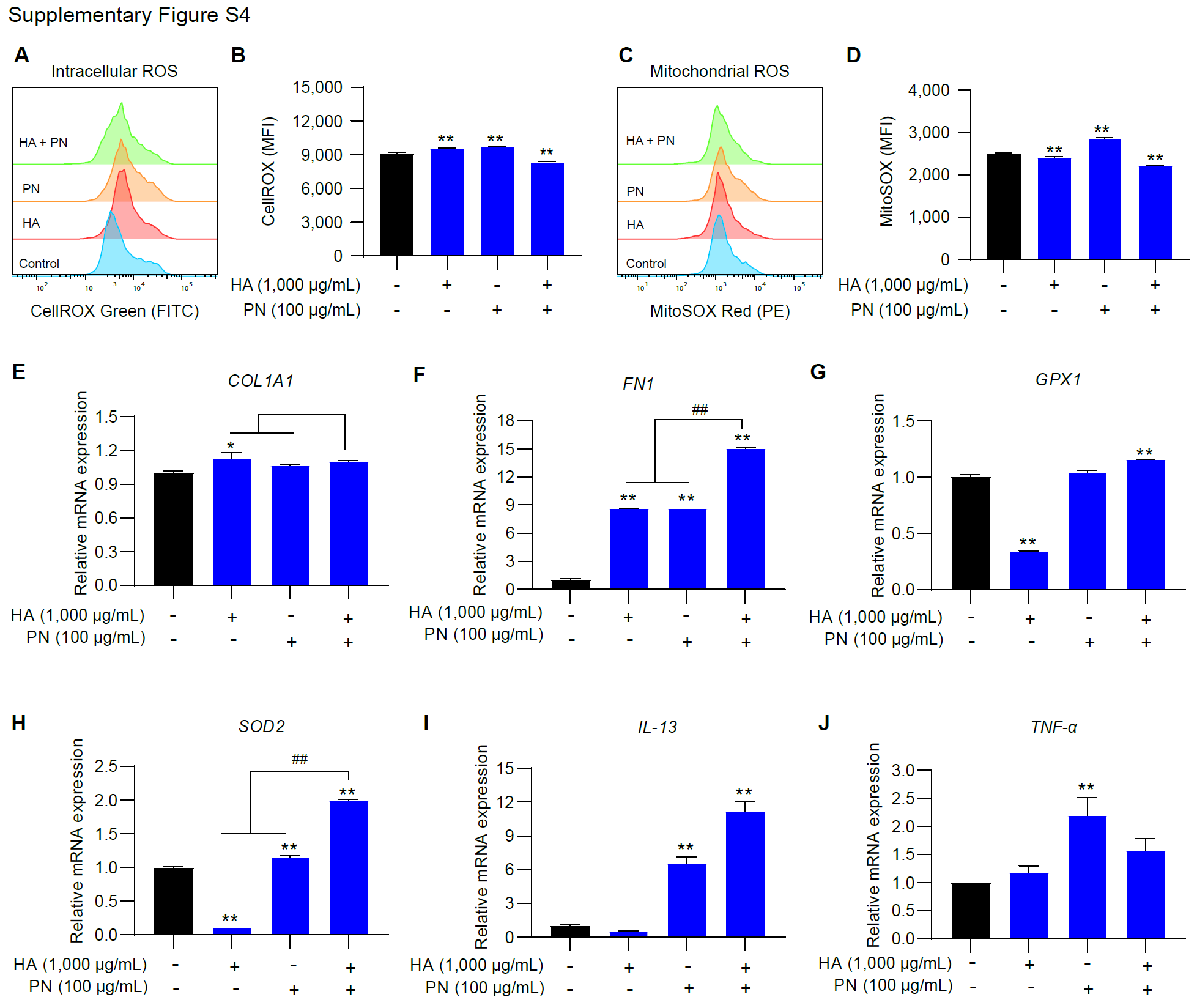
**

**Supplementary Figure S4. Baseline effects of HA and PN in HDFs without UVA irradiation.** (**A–D**) Flow cytometry analysis of intracellular and mitochondrial ROS after treatment with HA (1,000 µg/mL) and/or PN (100 µg/mL). (**E–J**) Relative mRNA expression of *COL1A1*, *FN1*, *GPX1, SOD2*, *IL-13*, and *TNF-α*. HA and PN did not elevate ROS levels and instead enhanced ECM- and antioxidant-related gene mRNA expression, as well as modulated cytokine mRNA expression profiles, indicating a favorable safety profile in the absence of UVA stress. Data are shown as mean ± SEM. (A–D, n = 5; E-J, n = 3). ^*^*p* < 0.05, ^**^*p* < 0.01 vs. control; ^##^*p* < 0.01 between indicated groups. Abbreviations: HA, hyaluronic acid; PN, polynucleotide; HDF, human dermal fibroblasts; UVA, ultraviolet A; COL1A1, collagen type I alpha 1 chain; FN1, fibronectin 1; GPX1, glutathione peroxidase 1; SOD2, superoxide dismutase 2; IL-13, interleukin-13; TNF-α, tumor necrosis factor-alpha; ROS, reactive oxygen species.

**
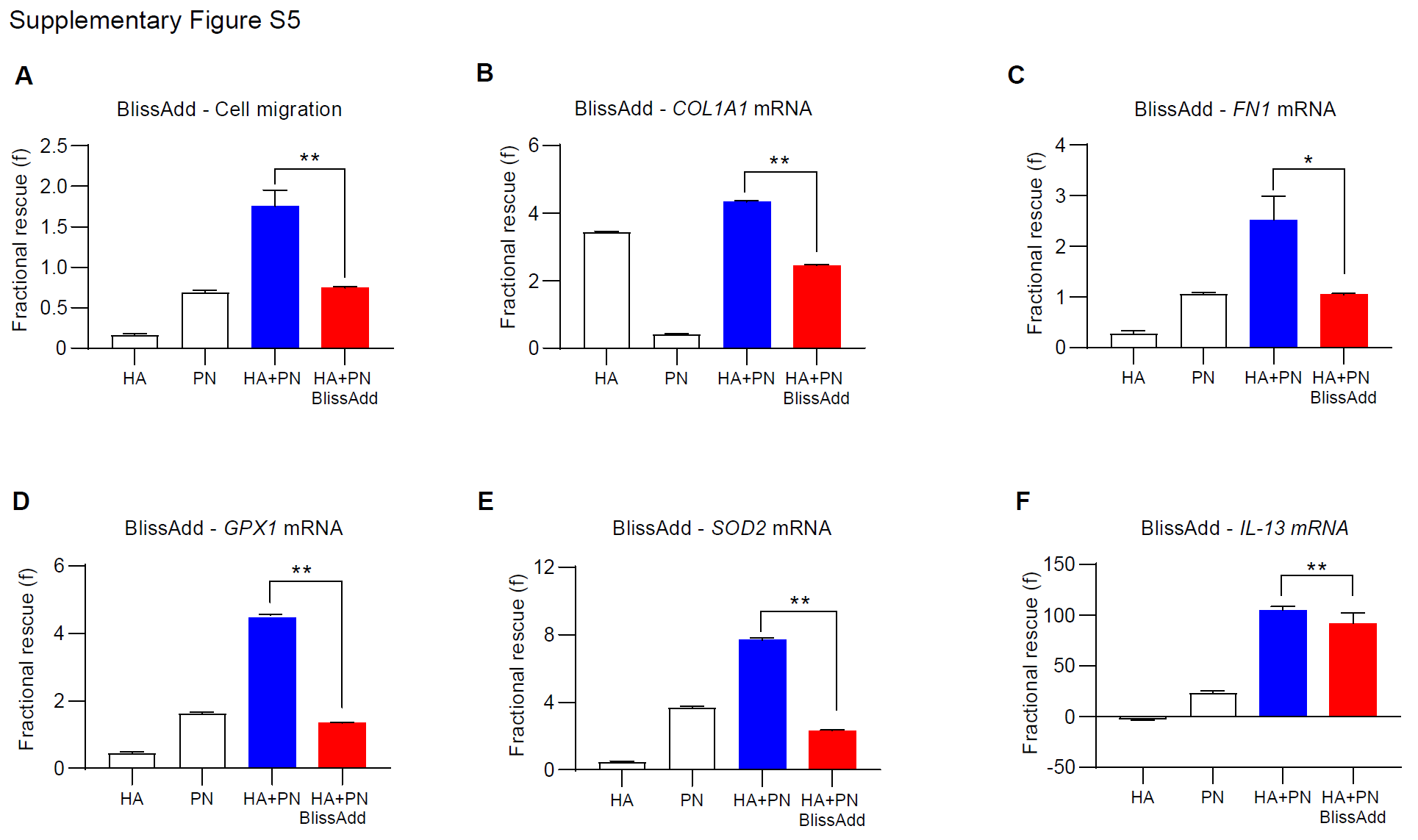
**

**Supplementary Figure S5. Bliss independence analysis confirming the synergistic effects of HA and PN.** The Bliss independence model was applied to quantify synergistic effects using the data from the migration assay (**A**; Fig. 4E) and the gene expression analyses (**B**, *COL1A1*; **C**, *FN1*; **D**, *GPX1*; **E**, S*OD2*; and **F**, *IL-13*), derived from the results shown in Fig. 5E and 5G–I. The predicted additive effect (*F*ₚᵣₑd = *F*_HA_ + *F*_PN_ – *F*_HA_ × *F*_PN_) was compared with the observed combined response (*F*ₒᵦₛ). Positive deviations (*F*ₒᵦₛ > *F*ₚᵣₑd or *F*ₒᵦₛ / *F*ₚᵣₑd > 1) indicate synergistic interaction, whereas values close to 1 represent additive effects. Bars represent mean ± SEM from three independent experiments (*n* = 3). *P* values were determined by one-sample, two-sided *t*-tests against the theoretical additive value (*p* < 0.05 considered significant). Abbreviations: HA, hyaluronic acid; PN, polynucleotide; COL1A1, collagen type I alpha 1 chain; FN1, fibronectin 1; GPX1, glutathione peroxidase 1; SOD2, superoxide dismutase 2; IL-13, interleukin-13.


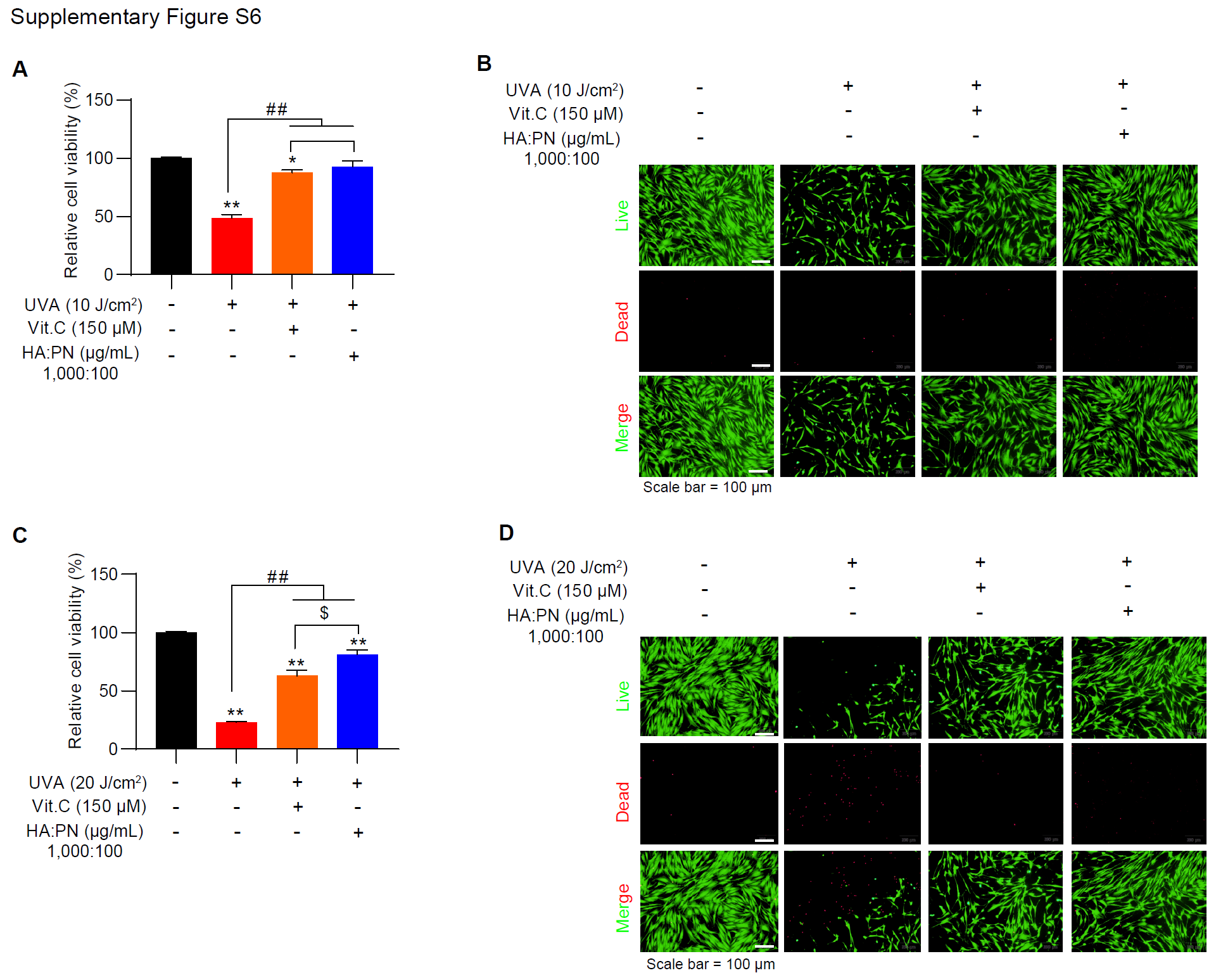


**Supplementary Figure S6. Comparative antioxidant effects of HA+PN and vitamin C under UVA exposure.** (**A**) Cell viability of HDFs assessed by CCK-8 assay after UVA irradiation at 10 J/cm² followed by treatment with 150 µM vitamin C (positive control) or HA+PN (HA:PN = 1,000 μg/mL:100 μg/mL). (**B**) Representative Live/Dead fluorescence images under the same conditions. (**C**) Cell viability of HDFs assessed by CCK-8 assay after UVA irradiation at 20 J/cm² followed by treatment with 150 µM vitamin C (positive control) or HA+PN (HA:PN = 1,000 μg/mL:100 μg/mL). (**D**) Representative Live/Dead fluorescence images under the same conditions. Data are shown as mean ± SEM. (A, C, n = 6). ^*^*p* < 0.05, ^**^*p* < 0.01 vs. control; ^##^*p* < 0.01, ^$^*p* < 0.05 between indicated groups. Abbreviations: HA, hyaluronic acid; PN, polynucleotide; UVA, ultraviolet A.

**Supplementary Table**

**Supplementary Table S1. Primer sequences for qPCR**

| **Primer** | **Sequence** |
| --- | --- |
| Human GAPDH | F: 5’-TTCGACAGTCAGCCGCATCTTCT-3’  R: 5’-AGGCGCCCAATACGACCAAATC-3’ |
| Human TNF-α | F: 5’-CGGAGGCTGAACAATAGGCT-3’  R: 5’-GGCCTCAGCAATGAGTGACA-3’ |
| Human IL-13 | F: 5’-GGAATCCCTGATCAACGTGT-3’  R: 5’-CCACCTCGATTTTGGTGTCT-3’ |
| Human SOD2 | F: 5’-TGGGGTAACTTAGCAGTTTC-3’  R: 5’-TCAGTCTCCAAAGTTTTTCC-3’ |
| Human GPX1 | F: 5’-GCGGGGCAAGGTACTACTTA-3’  R: 5’-TCTTGGCGTTCTCCTGATGC-3’ |
| Human COL1A1 | F: 5’-CCTGGATGCCATCAAAGTCT-3’  R: 5’-AATCCATCGGTCATGCTCTC-3’ |
| Human FN1 | F: 5’-CAGTGGGAGACCTCGAGAAG-3’  R: 5’-TCCCTCGGAACATCAGAAAC-3’ |
